# Effects of including sesame cake in broiler chicken feed: Performance, nutrient metabolizability, blood parameters, bone assessment, and profitability

**DOI:** 10.1016/j.psj.2025.105842

**Published:** 2025-09-11

**Authors:** Dayane Albuquerque da Silva, Maria do Carmo Mohaupt Marques Ludke, Jorge Vitor Ludke, Lilian Francisco Arantes de Souza, Mércia Rodrigues Barros, Ana Carolina Ferreira dos Santos, Fabiano Séllos Costa, Sérgio Peres, Camila Guedes Valadares, Arlei Coldebella, Esterfani Pereira da Silva, Lucas Rannier Ribeiro Antonino Carvalho, Apolônio Gomes Ribeiro

**Affiliations:** a Federal Rural University of Pernambuco, Department of Animal Science, Recife, Pernambuco, Brazil; b Embrapa Poultry and Swine, Concordia, Santa Catarina, Brazil; c University of Pernambuco, Mechanical Engineering Department, Recife, Pernambuco, Brazil; d Department of Physiology and Pharmacology, Karolinska Institutet, Biomedicum 5B, Solnavägen 9, S-171 77, Stockholm, Sweden; e Federal University of Paraíba, Department of Animal Science, Areia, Paraíba, Brazil

**Keywords:** Broiler chicken, Co-product, Nutraceutical, Performance, *Sesamum indicum*

## Abstract

This study investigated the use of sesame cake in broiler chicken feed, evaluating its impact on performance, nutrient metabolizability, and carcass and organ yield. Two hundred and forty Ross 308 line 1-day-old chicks weighing an average of 42 g ± 2 g were randomly distributed into four treatments with five replicates of 12 birds each. The treatments consisted of one control diet composed of corn, soybean meal, and meat and bone meal, in addition to three diets with sesame cake inclusions of 50, 100, and 150 g/kg. Data were statistically analyzed by using ANOVA, and later the Dunnett test (P<0.05) and linear and non-linear regression were performed. In the 1-to-7-day phase, there was a quadratic effect on live weight (LW), weight gain (WG) and feed conversion (FC), with the lowest estimated performance at approximately 95 g/kg of sesame cake (SC). Between 1–21 days, better performance was observed, with 50 g/kg of SC, with the highest estimated LW and WG at 56.85 g/kg, while feed intake showed a decreasing linear effect. In the 1-to-35-day and 1-to-42-day phases, performance did not differ from the control diet. In the 1-to-7-day phase, there was a linear reduction in the apparent metabolizability coefficients of gross energy (AMCGE), apparent metabolizable energy (AME) and metabolizable energy corrected for nitrogen (AMEn). From 8-21 days, the inclusion of 50 g/kg sesame cake caused a quadratic increase in the dry matter coefficients (AMCDM), AMCGE, crude protein (AMCCP), AME and AMEn, while 100 g/kg resulted in a decrease in AMCGE and AMCCP. In the 22-to-35-day phase, there was a linear decrease for AMCDM, AMCGE, AMCCP, AME and AMEn, and in the 36-to-42-day phase, a decrease with a quadratic effect was observed for AMCDM, AMCGE, AMCCP, AME and AMEn. The inclusion of sesame cake in the diets resulted in a linear increase in gizzard weight and a linear decrease in the plantar pad score. The cake also led to a linear reduction in leukocytes, lymphocytes, monocytes, creatinine, and HDL, and an increasing quadratic effect on heterophils. Although SC did not alter bone density, it reduced calcium concentration and the Ca:P ratio in the tibia (linear effect), while phosphorus had a quadratic response (peak at 57.83 g/kg). Economically, the return on investment (ROI) increased with the inclusion of SC, reaching 65% with 150 g/kg, evidencing economic viability associated with the use of the co-product. Sesame cake can be used in concentrations up to 56.85 g/kg in the initial phases, however the inclusion of up to 150 g/kg did not negatively affect performance in the total period of 1 to 42 days, nor bone densitometry parameters, and promoted good results in terms of health and profitability. Therefore, SC can be used up to 150 g/kg for broiler chickens. As SC is an alternative feed, it should be further studied in order to better understand its nutraceutical benefits.

## Introduction

Sesame cake (SC) is the main co-product resulting from the extraction of oil from sesame seeds. It is generally discarded, resulting in a waste of resources and economic losses (Wan et al., 2023). This co-product is characterized as a protein food, despite having high levels of ether extract, which also makes it an excellent source of lipids (Sá et al., 2022). Considering poultry production where the basic protein source is soybean meal, SC is starting to arouse academic and industrial interest with in view of the possible replacement of soybean meal, which would reduce commercial dependence on this major commodity.

In Brazil, SC is sold by agricultural associations and industries that process sesame seeds for oil extraction. Currently, a kilogram of SC is sold for R$1.80 (one real and eighty cents), equivalent to ₵0.30 (thirty US cents). SC is purchased at the price of 45 % soybean meal, of which one kilogram is sold at approximately R$2.64 (two reais and sixty-four cents) (National Supply Company, 2024) equivalent to ₵0.44 (forty-seven US cents), although this value is subject to variations throughout the harvest. SC has the advantage of being sold at a lower price in the Brazilian and certainly international market, in addition to having greater viability and a more stable and even fixed commercial value, and brings a greater economic return, as it is not a commodity.

In addition to the more attractive commercial value, SC has a high crude protein content (34 %) and a high fat content, which can reach up to 34.7 % of ether extract (Silva, 2015; Sharma et al., 2021). According to Primo (2008), when analyzing the amino acid composition of SC and comparing it with 45 % soybean meal, similar values were found for methionine, cystine, methionine + cystine, and threonine, with concentrations of 0.87 %, 0.6 %, 1.48 %, and 1.22 %, respectively. In terms of digestible amino acids, SC has a higher content of methionine (0.93 %) and arginine (3.74 %) compared to soybean meal, while the levels of the other amino acids are similar or lower (Primo, 2008; Heuzé et al., 2023).

Due to the protein composition of SC, some studies have tested this co-product in poultry feed. When using SC as a replacement for soybean meal in diets for broilers, Rama Rao et al. (2008) observed a linear reduction in alkaline phosphatase activity and serum protein concentration as the amount of SC increased in the diet.

In another study, Primo (2008) found that the total replacement of whole soybean meal with SC improved bird performance and carcass yield, without increasing feed intake or abdominal fat. These results demonstrate the potential of SC to improve animal performance. However, there is still a lack of information on the effects of SC on nutrient metabolizability, economic impact, among other things, confirming a need for more research in this area.

Due to the lack of studies that provide more complete and in-depth answers on the use of SC in broiler chicken feed, the present study hypothesized to determine the amount of SC energy used by chickens and that its inclusion in the diets would bring improvements to performance, nutrient metabolizability, blood variables, and carcass and organ yield, since it is rich in protein, lipids, and bioactive compounds, improving the bioavailability of nutrients for birds and consequently improving chicken performance, and also bringing greater financial return. In view of the above, the objective was to evaluate the potential of SC in broiler chicken diets, investigating the impact of this food on the hypothesized aspects, seeking to establish the effectiveness of its inclusion in the diet of broiler chickens.

## Material and methods

### Experimental site and ethics committee

The procedure for conducting this study was approved by the Animal Use Ethics Committee (CEUA) of the Federal Rural University of Pernambuco (UFRPE), under license number 7177220921.

### Obtaining sesame cake

The SC used in the study was acquired through the Residents Association of Ouricuri- Pernambuco, Brazil. To obtain the SC, the golden variety sesame seeds were cold pressed using a mechanical press (Model YZYX90, Manufacturer: Zhengzhou Sinoder Indutech Machinery Co., Ltd, Zhengzhou, Henan, China). The grains were pressed raw, eliminating the need for prior roasting, resulting in the obtaining of raw SC.

Subsequently, a bromatological analysis of SC was conducted (Table 1), covering parameters such as dry matter, crude protein, ash, calcium, phosphorus, and ether extract, following the AOAC specifications (Association of Official Analytical Chemistry, 2023). Gross energy was determined by using a calorimetric bomb (Model IKA C200, Manufacturer: IKA, Staufen, Breisgau, Germany). The bromatological analyses were performed at the Animal Nutrition Laboratory of UFRPE, and the energy determination was performed at the Fuel Laboratory of the University of Pernambuco (UPE).

**Table 1:** Bromatological composition of sesame cake and soybean meal used in the formulation of the experimental diet expressed in dry matter.

Nutritional Composition (g/kg)	Sesame Cake	Soybean Meal ⁎⁎
Dry Matter	939.20	896.00
Crude Protein	371.00	454.00
Ethereal Extract	324.70	19.50
Gross Energy (kcal/kg)	5796	4118
Metabolizable Energy for Poultry (kcal/kg)	4342	2258
Density (kg/m^3^)	560.00	650.00
Minerals (g/kg)		
Ash	65.32	58.6
Calcium	20.04	3.10
Phosphorus	8.69	5.70
Total Amino Acids (g/kg)*		
Methionine	9.30	6.10
Cystine	7.60	6.70
Methionine + Cystine	16.60	12.80
Lysine	8.60	28.0
Threonine	11.70	17.8
Tryptophan	4.50	6.40
Isoleucine	12.70	21.30
Leucine	22.70	35.10
Valine	15.50	22.20
Histidine	9.60	12.00
Phenylalanine	15.50	23.40
Tyrosine	11.70	16.60
Fatty Acids (g/kg)		
Palmitic (C16:0)	31.85	2.24
Stearic (C18:0)	15.26	0.82
Oleic (C18:1, cis)	120.66	4.50
Linoleic (C18:2, cis)	154.33	10.42
Linolenic (C18:3)	0.94	1.46
Arachidic (C20:0)	1.17	0.07
Eicosanoic (C20:1)	0.52	0.06
Iodine value (I_2_g/100 g oil)	37.41	2.52
Acidity (%)	0.45	0.006

⁎ The amino acid composition of SC is according to Heuzé et al. (2023).

⁎⁎ Composition of Soybean Meal according to Rostagno et al., 2017; soybean meal fatty acid values were estimated based on the composition of refined soybean oil (AOCS, 2013) adjusted to the ether extract value of the meal.

Fatty acid analysis (Table 1) was also performed by using gas chromatography of fatty acid methyl esters (Model GC 2010 AF, Manufacturer: Shimadzu, Nakagyo-ku, Kyoto, Japan), performed at the Separations Engineering Laboratory of the University of São Paulo (USP). The SC amino acid values used for feed formulation (Table 1) were in accordance with the work of.

For the use of sesame cake in the diets, the previously analyzed bromatological values were considered, in addition to the value of the apparent metabolizable energy corrected for the nitrogen balance of the sesame cake for broilers (4342 kcal/kg) previously determined by the total excreta collection method (Table 1).

The antinutritional factors of SC (Table 2) were determined according to the methodologies of Paiva e Coelho (1992); Amorim et al. (2008) and Bradford (1976). The antinutritional factors determined were the identification of lectins through the hemagglutination assay (Paiva e Coelho, 1992), the quantification of tannins (Amorim et al., 2008), the protease inhibitors through the trypsin activity inhibition assay (Bradford, 1976) and the determination of hemolytic saponins (Bradford, 1976). The analyses were performed at the Protein Biochemistry Laboratory of UFPE.

**Table 2:** Antinutritional factors present in sesame cake.

Compounds	Sesame Cake
Trypsin inhibitory activity (U/mg)	1.71 ± 0.231
Lectins (HA/ml)	-
Tannins (mg catechin/g)	0.20 ± 0.09
Hemolytic saponins (µg ES/mg)	243.77 ± 3.47
Total phenolic compounds (mg EAT/g)	2.03 ± 0.07
Residual phenolic compounds (mg EAT/g)	1.83 ± 0.09

TAE: tannic acid equivalents; SE: saponin equivalent; HA: hemagglutinating.

### Birds and bird housing

In this study, 240 one-day-old male chickens of the Ross 308 strain were used, with an average body weight of 42 ± 2 g. They were housed in a masonry shed, divided into polyvinyl chloride (PVC) box-shaped compartments measuring 2 × 1 m, lined with wood shavings bedding, and equipped with a tubular feeder and nipple drinker.

The temperature and relative humidity of the air were monitored by the thermohygrometer equipment (Model HM-01, Manufacturer: Highmed, São Paulo, Brazil) throughout the experimental period, obtaining the following averages 26.58 ± 1.30; 29.99 ± 1.60, and 22.74 ± 0.96 ºC for room temperature, maximum and minimum, respectively, and 73.82 ± 8.25; 89.79 ± 2.97 and 61.50 ± 8.18 % for room relative humidity, maximum and minimum, respectively. Due to the use of incandescent lamps in the heating system, 24-hour continuous lighting was implemented for the first seven days. From the eighth day of housing, a gradual reduction of thirty minutes in the daily duration of light occurred, eventually reaching 20 hours of light and 4 hours of darkness.

### Experimental design and experimental diets

The birds were distributed in a completely randomized design, consisting of 4 treatments with 5 replicates and 12 birds per experimental unit. The treatments consisted of a control diet based on corn, soybean meal, and meat and bone meal, and three test diets with inclusion of SC of 50, 100, and 150 g/kg (Tables 3 and 4). Diet formulation followed the nutritional guidelines of Rostagno et al. (2017) for male broilers with superior performance. Diets and water were made available *ad libitum*.

**Table 3:** Chemical composition and nutritional values of experimental diets in the pre-starter (1 to 7 days) and starter (8 to 21 days) phases.

	Sesame Cake Inclusion Levels (g/kg)							
Ingredients (g/kg)	1 to 7 days				8 to 21 days			
	0	50	100	150	0	50	100	150
Corn 78.6	431.56	440.89	450.21	447.76	458.84	468.16	477.48	486.80
Soybean, Meal 450	423.79	381.10	338.38	306.09	412.76	370.05	327.33	284.62
Sesame, Cake	0.00	50.00	100.00	150.00	0.00	50.00	100.00	150.00
Meat and Bone Meal 434.2	65.70	64.46	63.22	61.75	58.38	57.14	55.90	54.66
Soybean Oil	45.27	29.48	13.68	0.00	54.48	38.68	22.89	7.09
Wheat, Bran	17.79	17.79	17.79	17.79	0.00	0.00	0.00	0.00
Salt	4.29	4.28	4.27	4.26	4.27	4.26	4.25	4.24
Premix^1^	4.00	4.00	4.00	4.00	4.00	4.00	4.00	4.00
DL-Methionine 99 %	3.47	3.26	3.05	2.75	3.40	3.19	3.00	2.77
Calcitic Limestone	1.04	1.99	1.95	1.43	2.58	2.07	1.57	1.06
L-Lysine 78.8 %	2.97	2.46	2.94	3.58	0.91	1.85	2.80	3.75
Threonine 98.5 %	0.00	0.20	0.41	0.49	0.28	0.49	0.71	0.98
BHT^2^	0.10	0.10	0.10	0.10	0.10	0.10	0.10	0.10
Nutritional Composition (g/kg)								
AMEn (kcal/kg)	3000	3000	3000	3000	3100	3100	3100	3100
Crude Protein	250.00	250.00	250.00	250.00	241.50	241.50	241.50	241.50
Ethereal Extract	78.64	77.53	76.41	77.13	87.10	85.98	84.87	83.75
Linoleic Acid	28.27	26.83	25.38	25.14	32.72	31.27	29.83	28.38
Crude Fiber	29.82	29.66	29.49	29.63	28.07	27.90	27.74	27.57
Calcium	10.10	10.10	10.10	10.10	9.07	9.07	9.07	9.07
Available Phosphorus	4.82	4.82	4.82	4.82	4.32	4.32	4.32	4.32
Sodium	2.27	2.27	2.27	2.27	2.21	2.21	2.21	2.21
Digestible Amino Acids (g/kg)								
Lysine	13.60	13.60	13.60	13.60	13.06	13.06	13.06	13.06
Methionine + Cystine	9.89	9.89	9.89	9.89	9.66	9.66	9.66	9.66
Threonine	8.61	8.60	8.60	8.60	8.62	8.62	8.62	8.62
Tryptophan	2.83	2.78	2.73	2.74	2.74	2.69	2.64	2.59
Valine	10.75	10.63	10.51	10.55	10.41	10.29	10.17	10.05
Isoleucine	9.69	9.45	9.21	9.15	9.41	9.18	8.94	8.70
Leucine	18.55	18.28	18.00	17.94	18.18	17.91	17.63	17.36
Histidine	5.86	5.79	5.71	5.72	5.71	5.64	5.56	5.48
Analyzed Composition (g/kg)								
Dry Matter	881.11	880.91	888.82	889.26	890.41	891.36	890.10	892.20
Crude Protein	250.69	252.04	253.25	253.19	242.79	242.11	241.20	241.71
Gross Energy (kcal/kg)	4798	4287	4119	4174	4320	4164	4115	4159
Density (kg/m^3^)	671.61	661.69	651.75	642.10	686.01	676.08	666.15	656.21

1 Guaranteed levels per kilo of product: Iodine (minimum) 265.00 mg/kg; Selenium (minimum) 80.00 mg/kg; Copper (minimum) 3,000.00 mg/kg; Iron (minimum) 9,550.00 mg/kg; Manganese (minimum) 13.50 g/kg; Zinc (minimum) 12.50 g/kg; Folic Acid (minimum) 250.00 mg/kg; Niacin (minimum) 7,800.00 mg/kg; Biotin (minimum) 18.90 mg/kg; Pantothenic Acid (minimum) 3,180.00 mg/kg; Vitamin A (minimum) 2,400,000.00 IU/g (1 IU = 0.60 μg of ß-carotene); Vitamin B1 (minimum) 550.00 mg/kg; Vitamin B12 (minimum) 3.75 mg/kg; Vitamin B2 (minimum) 1,400.00 mg/kg; Vitamin B6 (minimum) 615.00 mg/kg; Vitamin D3 (minimum) 590,000.00 IU/g (1 IU= = 0.025 μg of cholecalciferol); Vitamin E (minimum) 4,250.00 IU/g (1 IU = 0.67 mg of d-α-tocopherol); Vitamin K (minimum) 875.00 mg/kg; Choline (minimum) 69.50 g/kg; BHT (minimum) 100.00 mg/kg; Halquinol (minimum) 7,500.00 mg/kg; ^2^ Antioxidant, Butyl-Hydroxytoluene.

**Table 4:** Chemical composition and nutritional values of experimental diets in the growth (22 to 35 days) and final (36 to 42 days) phases.

	Sesame Cake Inclusion Levels (g/kg)							
Ingredients (g/kg)	22 to 35 days 36 to 42 days				22 to 35 days 36 to 42 days			
	0	50	100	150	0	50	100	150
Corn 78.6	509.60	518.92	528.24	537.56	562.26	571.33	580.65	589.97
Soybean, Meal 450	369.96	327.24	284.53	241.82	333.31	290.98	248.27	205.55
Sesame, Cake	0.00	50.00	100.00	150.00	0.00	50.00	100.00	150.00
Meat and Bone, Meal 434.2	50.70	49.46	48.22	46.98	37.94	36.69	35.45	34.21
Soybean Oil	53.39	37.59	21.79	5.99	52.46	36.74	20.94	5.14
Salt	4.16	4.15	4.14	4.12	4.12	4.11	4.10	4.09
Premix^1^	4.00	4.00	4.00	4.00	4.00	4.00	4.00	4.00
DL-Methionine 99 %	3.26	3.05	2.83	2.62	2.33	2.12	1.91	1.69
Calcitic Limestone	2.99	2.48	1.98	1.47	2.85	2.34	1.83	1.33
L-Lysine 78.8 %	1.43	2.38	3.32	4.27	0.62	1.56	2.51	3.45
Threonine 98.5 %	0.43	0.64	0.85	1.06	0.00	0.03	0.24	0.46
BHT^2^	0.10	0.10	0.10	0.10	0.10	0.10	0.10	0.10
Nutritional Composition (g/kg)								
AMEn (kcal/kg)	3150	3150	3150	3150	3200	3200	3200	3200
Crude Protein	224.00	224.00	224.00	224.00	205.00	205.00	205.00	205.00
Ethereal Extract	86.15	85.03	83.92	82.80	84.93	83.88	82.76	81.65
Linoleic Acid	31.74	30.30	28.85	27.41	30.89	29.49	28.05	26.60
Crude Fiber	26.86	26.69	26.53	26.36	25.98	25.83	25.67	25.50
Calcium	8.22	8.22	8.22	8.22	6.61	6.61	6.61	6.61
Available Phosphorus	3.84	3.84	3.84	3.84	3.09	3.09	3.09	3.09
Sodium	2.11	2.11	2.11	2.11	2.01	2.01	2.01	2.01
Digestible Amino Acids (g/kg)								
Lysine	12.35	12.35	12.35	12.35	10.67	10.67	10.67	10.67
Methionine + Cystine	9.14	9.14	9.14	9.14	7.90	7.90	7.90	7.90
Threonine	8.15	8.15	8.15	8.15	7.20	7.04	7.04	7.04
Tryptophan	2.51	2.46	2.41	2.36	2.31	2.26	2.21	2.17
Valine	9.61	9.49	9.37	9.25	8.85	8.74	8.62	8.50
Isoleucine	8.66	8.42	8.19	7.95	7.98	7.75	7.52	7.28
Leucine	17.16	16.89	16.61	16.34	16.26	15.99	15.72	15.44
Histidine	5.33	5.25	5.18	5.10	4.99	4.92	4.84	4.76
Analyzed Composition (g/kg)								
Dry Matter	908.80	902.47	908.84	904.43	904.54	905.10	907.41	906.04
Crude Protein	223.73	221.19	228.31	225.78	205.53	202.12	204.49	203.28
Gross Energy (kcal/kg)	4611	4220	4221	4225	4406	4433	4246	4347
Density (kg/m^3^)	688.55	678.62	668.69	658.76	691.70	681.90	671.97	661.86

1 Guaranteed levels per kilo of product: Iodine (minimum) 265.00 mg/kg; Selenium (minimum) 80.00 mg/kg; Copper (minimum) 3,000.00 mg/kg; Iron (minimum) 9,550.00 mg/kg; Manganese (minimum) 13.50 g/kg; Zinc (minimum) 12.50 g/kg; Folic Acid (minimum) 250.00 mg/kg; Niacin (minimum) 7,800.00 mg/kg; Biotin (minimum) 18.90 mg/kg; Pantothenic Acid (minimum) 3,180.00 mg/kg; Vitamin A (minimum) 2,400,000.00 IU/g (1 IU = 0.60 μg of ß-carotene); Vitamin B1 (minimum) 550.00 mg/kg; Vitamin B12 (minimum) 3.75 mg/kg; Vitamin B2 (minimum) 1,400.00 mg/kg; Vitamin B6 (minimum) 615.00 mg/kg; Vitamin D3 (minimum) 590,000.00 IU/g (1 IU= = 0.025 μg of cholecalciferol); Vitamin E (minimum) 4,250.00 IU/g (1 IU = 0.67 mg of d-α-tocopherol); Vitamin K (minimum) 875.00 mg/kg; Choline (minimum) 69.50 g/kg; BHT (minimum) 100.00 mg/kg; Halquinol (minimum) 7,500.00 mg/kg; ^2^ Antioxidant, Butyl-Hydroxytoluene.

### Performance

The feeding program was divided into four phases: pre-starter (1-7 days), starter (8-21 days), growth (22-35 days) and finisher (36-42 days). In each phase, production data were recorded, including the weight of the experimental unit and feed intake (FI), with the aim of calculating weight gain (WG) and feed conversion (FC).

### Plantar pad assessment

**Fig. 1:**
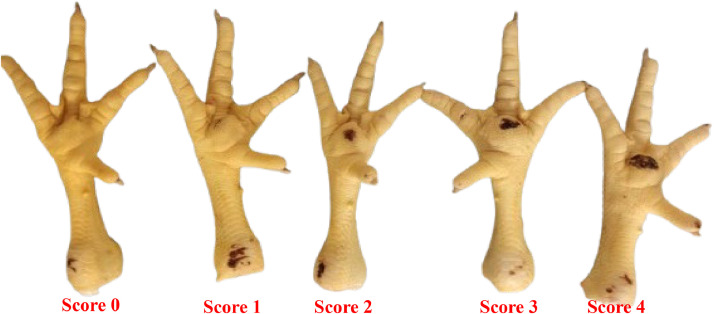
Plantar pads of the evaluated chickens, according to their scores.

At 42 days of age, the plantar pads of 10 birds per experimental unit were evaluated in order to determine the lesions caused by pododermatitis by using a 5-point scale, where: Score 0: intact plantar pad; Score 1: lesion covering less than 25 % of the affected pad; Score 2: lesion covering 26 to 50 % of the pad; Score 3: lesion covering 51 to 75 % of the pad; Score 4: lesion covering more than 76 % of the pad (Fig. 1). The methodology for evaluating the plantar pad was adapted from Mendes et al. (2012).

### Nutrient metabolizability

The metabolizability of the experimental diets was performed by the method of partial collection of excreta in each box, with the use of 1 % of insoluble acid ash indicator (Manufacturer: Labsynth Produtos para Laboratórios Ltda, Product CELITE 545, Diadema, São Paulo, Brazil) added to the diets. The birds underwent two days of adaptation to the diet with the addition of the indicator and two days of excreta collection within the pre-initial (on days 5 and 6 of age), initial (on days 18 and 19 of age), growth (on days 32 and 33 of age), and finishing (on days 39 and 40 of age) phases. The excreta were packaged in identified plastic bags and stored in a freezer at −20°C. Subsequently, the excreta were thawed, homogenized, pre-dried in a forced circulation oven at 55°C for 72 hours, and sent to the Animal Nutrition Laboratory of UFRPE.

Bromatological analyses of dry matter (DM) and crude protein (CP) of the diets and excreta were performed according to the AOAC (2023). Gross energy (GE) of the diets and excreta were calculated by using a calorimetric bomb (Model IKA C200, Manufacturer: IKA, Staufen, Breisgau, Germany), to determine the apparent metabolizability coefficients of dry matter (AMCDM), crude protein (AMCCP) and gross energy (AMCGE), as well as the apparent metabolizable energy (AME) and the energy corrected for nitrogen balance (AMEn), following the formulas described by Matterson et al. (1965) and Sakomura and Rostagno (2016).

### Blood collection

At the end of the experimental phase (42 days), blood was collected from the birds for hematological and serum biochemistry tests. For hematology, 3 birds were selected per treatment, where 4 ml of blood was collected from each bird to perform red blood cell, hemoglobin, hematocrit, platelet, leukocyte, heterophil, lymphocyte, monocyte, and eosinophil counts; and to quantify total plasma proteins. Red blood cell, leukocyte, and platelet counts were performed in a Neubauer chamber, after dilution with Natt-Herrick reagent. Hematocrit was determined by using the microcapillary method.

For serum biochemistry analysis, 5 birds per treatment were selected, and 4 ml of blood was collected from each bird after they were fasted for 8 hours, to measure serum levels of alkaline phosphatase (Bioclin Kit, Reference k224-2), albumin (Bioclin Kit, Reference k040-1), uric acid (Bioclin Kit, Reference k139-2), creatinine (Bioclin Kit, Reference k222-1), total protein (Bioclin Kit, Reference k031-1), globulin (total protein - albumin), gamma glutamyl transferase (Bioclin Kit, Reference k080-2), aspartate aminotransferase (Bioclin Kit, Reference k048-6), alanine transferase (Bioclin Kit, Reference k049-6), glucose (Bioclin Kit, Reference k082-2), total cholesterol (Bioclin Kit, Reference k083-2), HDL cholesterol (Bioclin Kit, Reference k015-1), VLDL cholesterol (triglycerides/5), LDL cholesterol (total cholesterol – cholesterol (HDL + VLDL)), and triglycerides (Bioclin Kit, Reference k117-2).

### Carcass and organ yield

At 42 days of age, 1 bird per plot was selected according to the average weight of the plot, stunned by electronarcosis, and euthanized, after having been fasted for 8 hours. The weight and yield of the carcass (without feet, head, and viscera), parts (breast, thigh + drumstick, back, and wings), viscera (heart, ventricle (gizzard), proventriculus, liver, spleen, bursa of Fabricius, pancreas, thymus, and length of intestine), and total fat (fat from the abdominal region plus fat around the gizzard) were evaluated.

The carcasses were subjected to a cooling process in a cold chamber at 4°C for 12 hours, followed by weighing on a scale with a precision of 0.01 g. The yield of the carcass and viscera was calculated in relation to the live weight of the bird at the time of slaughter, while the yield of the cuts was determined in relation to the total weight of the carcass.

### Densitometry, Seedor index, and mineral composition of the tibia

After evaluation of the chicken carcasses, the right tibias of 5 birds per treatment were selected for analysis of bone densitometry, Seedor index, and bone mineral composition. Bone densitometry was determined by computed tomography using a Hi-speed FX1 CT scanner (Manufacturer: General Electric, Melbourne, Florida). The bones were positioned side by side on the examination table, and cross-sectional images were obtained with 2 mm thick slices, reconstruction every 1 mm, 120 kV and automatic tube current. Bone density was calculated based on the mean attenuation of the pixels in the region of interest, expressed in Hounsfield units (HU).

**Fig. 2:**
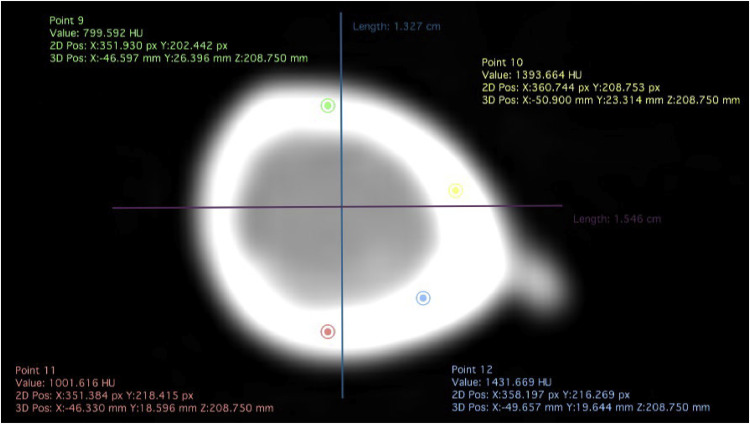
Cross-sectional tomographic image of the tibia of broiler chickens.

The images were analyzed by using Dicom software (Horos, version 1.1.7) to estimate bone radiodensity at three levels of the diaphysis (proximal, medial, and distal). Each region was divided into four quadrants, selecting a circular area for evaluation of the cortical bone (Fig. 2). The results were presented in Hounsfield units (HU), later corrected and converted to mg/cm^3^ of calcium hydroxyapatite, using the equation BMD = 200 HUt ∕ (HUw - HUb), according to the methodology described by Oliveira et al. (2012) and Simões de Souza et al. (2018).

To determine the Seedor index, the tibias were defleshed and pre-dried in a forced ventilation oven at 55°C for 48 hours. After drying, length measurements were recorded by using a digital caliper and the tibias were weighed to calculate the Seedor index, in order to provide an estimate of bone density (amount of minerals per unit area or volume), by means of the ratio of the ash weight (mg) to the length (mm) of the bone, according to Seedor et al. (1991).

For analysis of bone mineral composition, the tibias were calcined in a muffle furnace (Model 2000F, Manufacturer: Zezimaq, Belo Horizonte, Minas Gerais, Bazil) for 4 hours at 600°C (Yan et al., 2005). After determining the ashes, the calcium analysis was performed by atomic absorption spectrometry, according to analytical methods by flame atomic absorption spectrometry (Model K37-UVVIS, Manufacturer: Kasvi, Pinhais, Paraná, Brazil) and the phosphorus analysis by ultraviolet-visible spectrophotometry using the molybdovanadate reagent, according to Association of Official Analytical Chemists International (2012).

### Return on investment (ROI)

For the economic evaluation, the adjusted feed value in Brazilian reais (R$) for feed intake per treatment (R$/kg) and the sale price of live chicken (R$/kg) were considered. In this case, a value of R$5.50 (in US cents: ₵0.91) was assumed for each kilogram of live weight, along with the average weight gain per treatment. Then, the return on investment (ROI) was calculated as (revenue – costs) / investment, according to the formula established by Biocamp, a company specialized in animal nutrition. The revenue was determined by multiplying the average weight gain by the sales value of the bird per kg, and the investment was obtained by multiplying the total value of the feed by the intake. The prices of the ingredients that make up the feed are described in Table 5.

**Table 5:** Prices of ingredients used to calculate ROI.

Ingredients	Unit (kg)	Values/kg (R$)	Values/kg (US$)*
Corn 78.6	1	1.36	0.23
Soybean, Meal 450	1	2.64	0.64
Sesame, Cake	1	1.80	0.30
Meat and Bone, Meal 434.2	1	0.90	0.15
Soybean Oil	0.8	6.20	1.03
Salt	1	1.05	0.17
Premix	25	754.50	125.08
DL-Methionine	25	857.80	142.20
Limestone	50	15.00	2.49
L-Lysine	25	955.00	158.32
Threonine	25	539.00	89.35
BHT	25	338.25	56.07

⁎ The currency converter of the Central Bank of Brazil was used. 1 United States Dollar/USD (220) = 6.0322 Real/BRL (790).

### Statistical analysis

The data were analyzed by using the Statistical Analysis System (SAS) software version 9.4. Initially, the normality of the residuals was assessed by the Shapiro-Wilk test, and the homogeneity of the variances by the Levene test. To compare the effects of the treatments, a univariate Analysis of Variance (ANOVA) was performed, according to the mathematical model by the PROC GLM procedure of SAS, followed by the Dunnett test.

Furthermore, a regression analysis was performed to determine the best inclusion level of sesame cake (SC), considering the tested levels of 0, 50, 100, and 150 g/kg. The regression equations were adjusted according to the significance of the linear and quadratic coefficients, by using the PROC REG procedure of SAS.

Each treatment was tested with five replicates, and the results were expressed as mean ± standard deviation. The significance level adopted was 5 %.

Dunnett's analysis was performed using the following statistical model:

Yij=μ+Ti+ϵij

Where:

Yij = Observed value for treatment iii in repeat jjj.
μ= Overall average;
Ti= Effect of treatment iii (*i* = 1, 2, ...,5).
ϵij= Random error, assumed as ϵij∼N (0, σ2).

## Results

### Performance

**Fig. 3:**
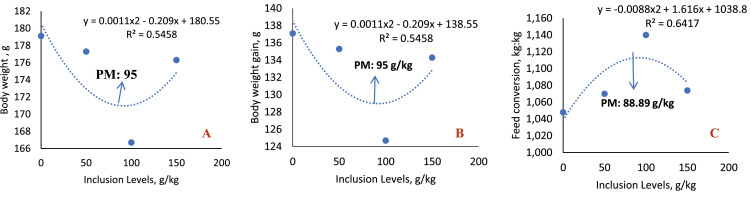
Quadratic effect of body weight (A), body weight gain (B), and feed conversion (C) of birds fed SC at ages 1-7 days.

In the 1-to-7-day phase (Table 6; Fig. 3), a significant effect was observed on the averages of live weight (*P* = 0.0085), weight gain (*P* < 0.0085), and feed conversion (*P* = 0.0089), with quadratic adjustment for all variables. The lowest live weight and weight gain were estimated with the inclusion of 95 g/kg of sesame cake, while the worst feed conversion occurred with 88.89 g/kg. By comparing the averages, only the 100 g/kg level differed significantly from the control diet.

**Table 6:** Average values of body weight (BW), body weight gain (BWG), feed intake (FI), and feed conversion (FC) of broilers fed diets with increasing levels of sesame cake.

Inclusion Levels (g/kg)	BW (g/bird)	WG (g/bird)	FI (g/bird)	FC (kg:kg)
	1 – 7 days			
0	179.1 ± 1.7	137.1 ± 1.7	143.6 ± 0.9	1.048 ± 0.0097
50	177.3 ± 4.1	135.3 ± 4.1	144.8 ± 5.2	1.070±0.0172
100	166.7 ± 0.3*	124.7 ± 0.3*	142.1 ± 2.6	1.140 ± 0.0202*
150	176.3 ± 1.6	134.3 ± 1.6	144.1 ± 1.3	1.074 ± 0.0189
Mean	174.84	132.84	143.67	1.0830
SEM	1.5454	1.5454	1.3927	0.0111
p-value	0.0085	0.0085	0.9321	0.0089
Regression	< 0.0001⁎⁎⁎	< 0.0001⁎⁎⁎	NS	< 0.0001⁎⁎⁎
	1 – 21 days			
0	1158 ± 9	1116 ± 9	1354 ± 24	1.213 ± 0.0245
50	1200 ± 4*	1158 ± 4*	1353 ± 22	1.168 ± 0.0172
100	1161 ± 5	1119 ± 5	1333 ± 16	1.192 ± 0.0119
150	1122 ± 3*	1080 ± 3*	1274 ± 5*	1.181 ± 0.0042
Mean	1160.16	1118.16	1328.71	1.1885
SEM	6.8569	6.8569	11.1737	0.0084
p-value	<0.0001	<0.0001	0.0222	0.2864
Regression	< 0.0001⁎⁎⁎	< 0.0001⁎⁎⁎	0.0058⁎⁎	NS
	1 – 35 days			
0	2885 ± 25	2843 ± 25	3841 ± 50	1.351 ± 0.0141
50	2889 ± 41	2847 ± 41	3807 ± 64	1.338 ± 0.0219
100	2820 ± 39	2778 ± 39	3779 ± 36	1.361 ± 0.0118
150	2772 ± 43	2730 ± 43	3702 ± 29	1.357 ± 0.0147
Mean	2841.65	2799.65	3782.16	1.3515
SEM	20.6868	20.6868	24.6242	0.0076
p-value	0.1289	0.1289	0.2293	0.7683
Regression	NS	NS	NS	NS
	1 – 42 days			
0	3681 ± 24	3639 ± 24	5383 ± 59	1.479 ± 0.0114
50	3705 ± 42	3663 ± 42	5360 ± 82	1.464 ± 0.0219
100	3612 ± 47	3570 ± 47	5322 ± 52	1.491 ± 0.0101
150	3606 ± 41	3564 ± 41	5267 ± 38	1.478 ± 0.0129
Mean	3650.80	3608.80	5332.95	1.4781
SEM	20.6421	20.6421	29.2784	0.0072
p-value	0.2336	0.2336	0.5556	0.6363
Regression	NS	NS	NS	NS

⁎ Statistical difference (Dunnett test).

⁎⁎ Linear effect.

⁎⁎⁎ Quadratic effect. Linear Effect: (Feed Intake 1 to 21: *y*= −0.52x + 1367.5, R^2^= 0.797). Quadratic Effect: (Live weight 1 to 7: *y* = 0.0011×^2^ – 0.209x + 180.55; R^2^= 0.5458; Minimum Point: 95 g/kg); (Live weight 1 to 21: *y*= −0.0081×^2^ + 0.921x + 1162.1; R^2^= 0.8924; Maximum Point: 56.85 g/kg); (Weight Gain 1 to 7: *y* = 0.0011×^2^ – 0.209x + 138.55; R^2^= 0.5458; Minimum Point: 95 g/kg); (Weight Gain 1 to 21: *y*= −0.0081×^2^ + 0.921x + 1120.1; R^2^= 0.8924; Maximum Point: 56.85 g/kg); (Feed Conversion 1 a 7: *y*= −9E-06×^2^ + 0.0016x + 1.0388; R^2^= 0.6417; Maximum Point: 88.89 g/kg). SEM: Standard Error of the Mean.

**Fig. 4:**
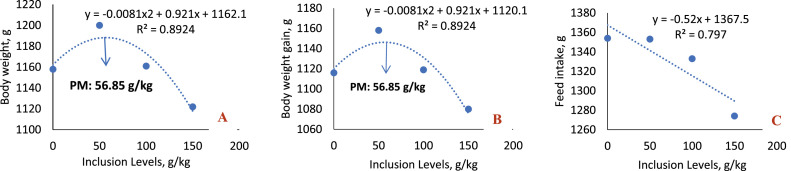
Linear and quadratic effect of body weight (A), body weight gain (B) and feed intake (C) of birds fed SC at ages 1-21 days.

In the 1-to-21-day phase (Table 6; Fig. 4), significant differences were observed in the values of live weight (*P* < 0.0001), weight gain (*P* < 0.0001) and feed intake (*P* = 0.0222). There was a quadratic effect for live weight and weight gain (*P* < 0.0001 for both), and a decreasing linear effect for feed intake (*P* = 0.0058), as the inclusion levels of sesame cake increased. The best results for live weight and weight gain were obtained with the inclusion of 50 g/kg of SC, with an estimated optimum point of 56.85 g/kg. In contrast, the maximum level tested (150 g/kg) compromised the performance of the broilers.

In the other phases, from 1 to 35 and from 1 to 42 days (Table 6), no statistical differences were observed between treatments for the variables of live weight, weight gain, feed intake, and feed conversion. Thus, the performance of the chickens fed with different levels of sesame cake was similar to that of the chickens that received the control diet, indicating that the inclusion of the by-product did not compromise the development of the birds throughout the total rearing period.

### Plantar pad assessment

**Fig. 5:**
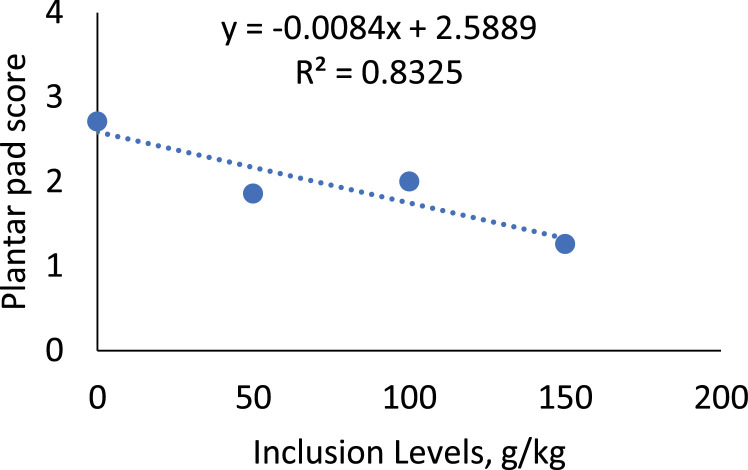
Linear effect of the plantar pad score of birds fed with SC.

The plantar pad of the chickens (Table 7; Fig. 5) showed a statistical difference between the treatments (*P* = 0.0173), with a decreasing linear effect (*P* = 0.0039), indicating a progressive improvement in the score as the inclusion of sesame cake in the diet increased, reaching a score of 1 at the level of 150 g/kg.

**Table 7:** Average score values for the evaluation of the plantar pad of broiler chickens fed with the inclusion of increasing levels of sesame cake.

Inclusion Levels (g/kg)	Score	SEM	*p-value*	Regression
0	3 ± 0.322	0.1740	0.0173	0.0039⁎⁎
50	2 ± 0.163			
100	2 ± 0.362			
150	1 ± 0.223*			
Mean	2			

⁎ Statistical difference (Dunnett test).

⁎⁎ Linear effect. ***Quadratic effect. Linear Effect (*y* = −8.402x + 2588.9, R² = 0.8325). SEM: Standard Error of the Mean.

### Nutrient metabolizability

**Fig. 6:**
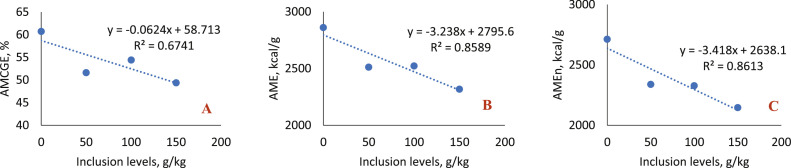
Linear effect of the metabolizability coefficients of gross energy (A), apparent metabolizable energy (B) and apparent metabolizable energy corrected for nitrogen balance (C) of birds fed with SC, in the 1–7-day phase.

In the 1-to-7-day phase (Table 8; Fig. 6), the inclusion of sesame cake resulted in a significant reduction in the apparent metabolizability coefficients of gross energy (AMCGE), apparent metabolizable energy (AME) and apparent metabolizable energy corrected for nitrogen (AMEn), with all inclusion levels differing statistically from the control diet and presenting a decreasing linear effect (*P* < 0.0001 for all variables).

**Table 8:** Average values of the apparent metabolizability coefficients of nutrients, dry matter (AMCDM), gross energy (AMCGE), crude protein (AMCCP) and apparent metabolizable energy (AME) and apparent energy corrected for nitrogen (AMEn), of broilers fed diets with increasing levels of sesame cake.

Inclusion Levels (g/kg)	1 – 7 days				
	AMCDM	AMCGE	AMCCP	AME	AMEn
0	44.97± 2.23	60.71± 1.66	49.72± 2.64	2860± 47	2713± 42
50	41.53± 0.70	51.62± 0.48*	46.04± 2.59	2512± 23*	2339± 32*
100	47.90± 0.54	54.42± 0.60*	51.80± 1.41	2522± 28*	2328± 27*
150	43.27± 0.26	49.37± 0.60*	45.35± 2.49	2317± 28*	2147± 32*
Mean	43.38	53.11	48.54	2502	2314
SEM	2.6936	2.1552	5.2272	73.8080	75.7406
*p-value*	0.1117	<0.0001	0.2053	<0.0001	<0.0001
Regression	NS	<0.0001⁎⁎	NS	<0.0001⁎⁎	<0.0001⁎⁎
	8 – 21 days				
	AMCDM	AMCGE	AMCCP	AME	AMEn
0	60.99± 0.43	68.60± 0.51	52.25± 0.42	3239± 16	3095± 14
50	64.17± 0.83*	74.40± 0.24*	56.47± 0.41*	3494± 21*	3302± 18*
100	62.17± 0.71	71.64± 0.61*	55.13± 0.36*	3312± 28	3153± 28
150	60.88± 0.72	68.18± 0.54	51.50± 1.15	3231± 13	3085± 13
Mean	62.05	70.71	53.84	3319	3159
SEM	1.2945	0.9270	4.6697	49.9584	49.9774
*p-value*	0.0130	<0.0001	0.0002	<0.0001	<0.0001
Regression	<0.0001⁎⁎⁎	<0.0001⁎⁎⁎	0.0018⁎⁎⁎	NS	NS
	22 – 35 days				
	AMCDM	AMCGE	AMCCP	AME	AMEn
0	79.73± 0.68	84.07± 0.54	77.38± 1.04	4221± 6	3974± 6
50	73.98± 0.76*	77.89± 0.80*	69.37± 1.63*	3642± 38*	3418± 33*
100	72.05± 0.35*	75.77± 0.31*	68.79± 1.56*	3519± 14*	3292± 15*
150	69.06± 0.76*	73.75± 0.72*	67.12± 1.11*	3445± 34*	3224± 32*
Mean	72.40	76.01	69.82	3552	3339
SEM	1.4715	1.3916	3.0362	59.1202	54.5870
*p-value*	<0.0001	<0.0001	0.0003	<0.0001	<0.0001
Regression	<0.0001⁎⁎	<0.0001⁎⁎	<0.0001⁎⁎	<0.0001⁎⁎	<0.0001⁎⁎
	36 – 42 days				
	AMCDM	AMCGE	AMCCP	AME	AMEn
0	78.13± 0.38	82.64± 0.42	71.73± 1.36	4026± 20	3811± 20
50	72.11± 0.97*	77.59± 0.88*	60.13± 1.06*	3800± 43*	3624± 40*
100	73.20± 0.57*	77.58± 0.45*	60.99± 1.44*	3630± 21*	3449± 17*
150	73.13± 0.82*	77.50± 0.68*	62.25± 3.09*	3718± 33*	3535± 26*
Mean	73.57	78.35	62.20	3764	3578
SEM	1.6107	1.4186	4.2673	68.6453	61.0970
*p-value*	<0.0001	<0.0001	0.0018	<0.0001	<0.0001
Regression	0.0008⁎⁎⁎	<0.0001⁎⁎	0.0039⁎⁎⁎	0.0001⁎⁎⁎	0.0001⁎⁎⁎

⁎ Statistical difference (Dunnett test).

⁎⁎ Linear effect.

⁎⁎⁎ Quadratic effect. Linear Effect: (AMCGE 1-7 days: *y*=−0.0624x + 58.713; R^2^= 0.6741); (AME 1-7 days: *y*=−3.238x + 2795.6; R^2^= 0.8589); (AMEn 1-7 days: *y*=−3.418x + 2638.1 R^2^= 0.8613); (AMCDM 22-35 days: *y*=−0.0679x + 78.796; R^2^= 0.949); (AMCGE 22-35 days: *y*=−0.0662x + 82.832; R^2^= 0.9146); (AMCCP 22-35 days: *y*=−0.0627x + 75.369; R^2^= 0.7824); (AME 22-35 days: *y*=−4.902x + 4074.4; R^2^=0.8066); (AMEn 22-35 days: *y*=−4.752x + 3833.4; R^2^= 0.8094); (AMCGE 36-42 days: *y*=−0.0309x + 81.142; R^2^=0.6141). Quadratic Effect: (AMCDM 8-21 days: *y*=−0.0004×^2^ + 0.0624x + 61.285; R^2^= 0.7522; Maximum Point: 78.00 g/kg); (AMCGE 8-21 days: *y*=−0.0009×^2^ + 0.1309x + 68.993; R^2^= 0.8781; Maximum Point: 72.72 g/kg); (AMCCP 8-21 days: *y*=−0.0008×2 + 0.1106x + 52.414; R2= 0.9678; Maximum Point: 69.13 g/kg); (AMCDM 36-42 days: *y* = 0.0006×2 – 0.1171x + 77.717; R2= 0.8442; Minimum Point: 97.58 g/kg); (AMCCP 36-42 days: *y* = 0.0013×2 – 0.2481x + 71.127; R2= 0.9161; Minimum Point: 95.42 g/kg); (AME 36-42 days: *y* = 0.0314×2 – 6.898x + 4036.1; R2= 0.9764; Minimum Point: 109.84 g/kg); (AMEn 36-42 days: *y* = 0.0273×2 – 6.101x + 3823.5; R2= 0.957; Minimum Point: 111.74 g/kg). SEM: Standard Error of the Mean.

**Fig. 7:**
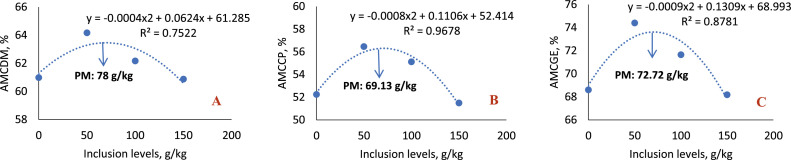
Quadratic effect of the metabolizability coefficients of dry matter (A), crude protein (B) and gross energy (C) of birds fed with SC, in the 8-21-day phase.

In the 8-to 21-day phase (Table 8; Fig. 7), statistical differences were observed for all variables analyzed (AMCDM, AMCGE, AMCCP, AME and AMEn) with the inclusion of 50 g/kg of sesame cake, indicating greater use of nutrients and energy at this level. At the 100 g/kg level, there was also an improvement in the AMCGE and AMCCP values, although lower than those obtained with 50 g/kg. Regression analysis indicated a quadratic effect for AMCDM (*P* < 0.0001), AMCCP (*P* = 0.0018) and AMCGE (*P* < 0.0001), with the highest estimated coefficients at the inclusion levels of 78.00 g/kg, 72.72 g/kg, and 69.13 g/kg, respectively, evidencing an optimal inclusion point for better use of nutrients and energy.

**Fig. 8:**
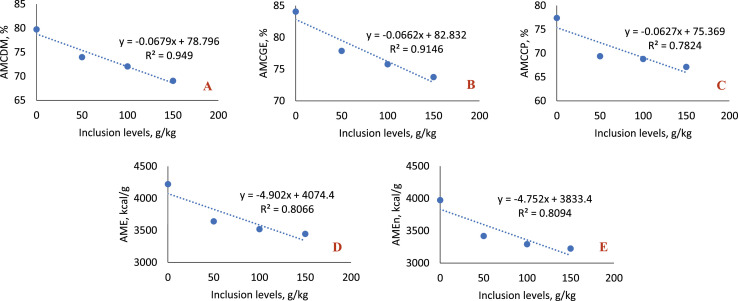
Linear effect of the metabolizability coefficients of dry matter (A), gross energy (B), crude protein (C), apparent metabolizable energy (D) and apparent metabolizable energy corrected for nitrogen balance (E) of birds fed with SC, in the 22-35 day phase.

In the 22-to-35-day phase (Table 8; Fig. 8), all variables analyzed showed statistical difference at all levels of sesame cake inclusion, with a gradual reduction in the use of nutrients and energy. This behavior reflected a significant decreasing linear effect for all variables (*P* < 0.0001).

**Fig. 9:**
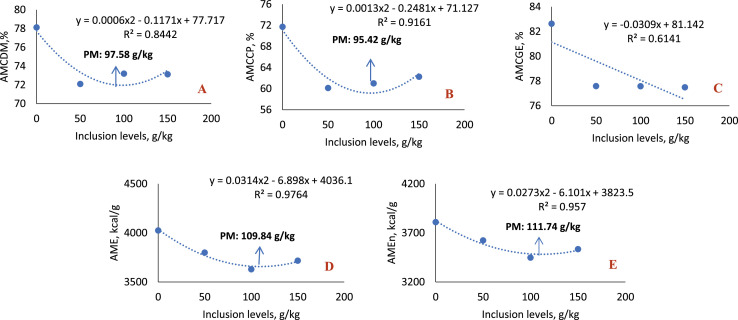
Linear and quadratic effect of the metabolizability coefficients of dry matter (A), crude protein (B), gross energy (C), apparent metabolizable energy (D) and apparent metabolizable energy corrected for nitrogen balance (E) of birds fed with SC, in the 36–42-day phase.

In the 36-to-42-day phase (Table 8; Fig. 9), a statistical difference was also observed at all inclusion levels for all variables evaluated. There was a reduction in the use of nutrients and energy, with a decreasing quadratic effect for AMCDM (*P* = 0.0008), AMCCP (*P* = 0.0039), AME (*P* = 0.0001) and AMEn (*P* = 0.0001), with the lowest coefficients estimated at the levels of 97.58 g/kg, 95.42 g/kg, 109.84 g/kg and 111.74 g/kg, respectively. The AMCGE variable showed a decreasing linear behavior (*P* < 0.0001).

### Blood collection

**Fig. 10:**
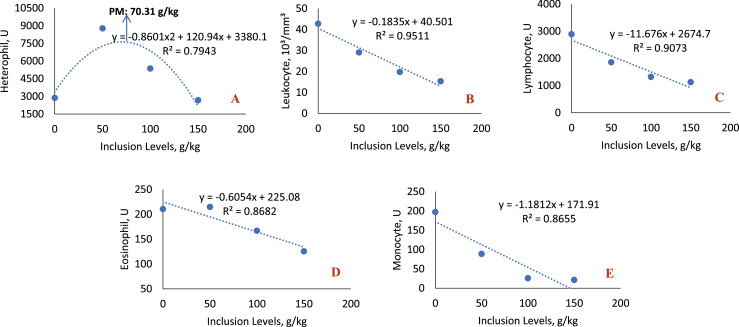
Quadratic effect of heterophil (A) and linear effect for leukocyte (B), lymphocyte (C), eosinophil (D), monocyte (E) of birds fed with SC.

**Fig. 11:**
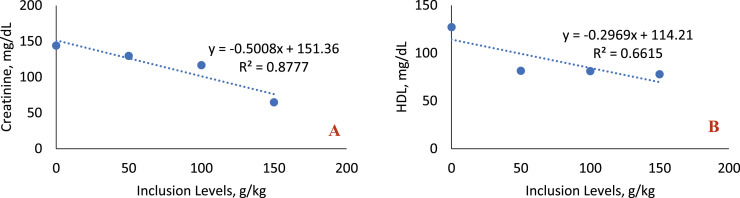
Linear effect of creatinine (A) and HDL (B) of birds fed with SC.

The inclusion of sesame cake in the diet caused a decrease in the mean values of some hematological variables (Table 9, Fig. 10), resulting in a linear decreasing trend of leukocytes (*P* = 0.0001) and lymphocytes (*P* < 0.0001). All diets containing sesame cake differed from the control diet. Regarding the heterophil value, it showed a quadratic effect (*P* = 0.0010). The level of 50 g/kg increased the circulating number of this defense cell, and the maximum point was 70.31 g/kg. Monocytes reduced linearly (*P* = 0.0002) with the inclusion of the cake, and all the means differed from the reference diet. In serum biochemistry (Table 10, Fig. 11), the inclusion of the cake resulted in a linear decrease (*P* = 0.0014) in serum creatinine values, which by the test of means, differed from the control diet only in the last level of cake. The mean values of HDL were reduced linearly (*P* = 0.0006) as the sesame cake was included in the diet, where all diets containing the sesame cake differed from the control diet.

**Table 9:** Mean hematological values of broiler chickens fed with increasing levels of sesame cake inclusion.

	Inclusion Levels (g/kg)							
Hematology	0	50	100	150	Mean	SEM	*p-value*	Regression
RBC,10^3^/mm^3^	3.17±0.92	5.34±0.53	4.07±0.47	4.70±1.08	4.32	0.4159	0.3212	NS
HETC, %	28.67±0.88	32.33±2.85	29.67±2.33	27.33±0.33	29.50	0.9809	0.3571	NS
HEMO, g/dL	9.57±0.30	10.80±0.95	9.90±0.76	9.10±0.10	9.84	0.3272	0.3381	NS
HETE, U	2880±931	8777±694*	5373±1E^3^	2669±571	4924.75	827.60	0.0032	0.0010⁎⁎⁎
LEUK,10^3^/mm^3^	42.80±4.30	29.08±3.18*	19.75±2.00*	15.33±2.04*	26.74	3.4243	0.0010	0.0001⁎⁎
LYMPH, U	2890±247	1861±198*	1321±52*	1124±163*	1799.00	220.42	0.0005	<0.0001⁎⁎
EOS, U	210.7 ± 30.0	215.0 ± 45.2	167.3 ± 4.7	125.7 ± 7.2	179.67	16.0375	0.1507	NS
MONO, U	197.3 ± 38.7	88.67±12.45*	26.00±5.29*	21.33±7.13*	83.33	23.1675	0.0011	0.0002⁎⁎
PLAT, mm^3^	103.5 ± 0.5	114.3 ± 3.7	105.8 ± 9.5	97.73±9.40	105.34	3.4567	0.4487	NS
TPP, g/dL	3.33±0.29	3.23±0.15	3.27±0.27	3.03±0.09	3.22	0.0968	0.7782	NS
MRCV, fL	80.03±7.54	62.23±9.79	73.70±3.84	54.53±6.36	67.63	4.2821	0.1324	NS

⁎ Statistical difference (Dunnett test).

⁎⁎ Linear effect.

⁎⁎⁎ Quadratic effect. Linear Effect (LEUC: *y* = −0.1835x + 40.501, R² = 0.9511); (LYMPH: *y*= −11.676x + 2674.7; R² = 0.9073); (MONO: *y* = −1.1812x + 171.91, R² = 0.8655). Quadratic Effect (HETE: *y* = −0.8601×2 + 120.94x + 3380.1, R² = 0.7943, Maximum Point: 70.31 g/kg). RBC: Red blood cells; HETC: Hematocrit; HEMO: Hemoglobin; HETE: Heterophil; LEUK: Leukocyte; LYMPH: Lymphocyte; EOS: Eosinophil; MONO: Monocyte; PLAT: Platelets; TPP: Total Plasma Proteins; MRCV: Mean Red Cell Volume. SEM: Standard Error of the Mean. dL: Deciliter; fL: Fentoliter; g: Grams; mm: Millimeter; U: Unit. NS: Not significant.

**Table 10:** Mean serum biochemistry values of broilers fed with the inclusion of increasing levels of sesame cake.

	Inclusion Levels (g/kg)							
Serum Biochemistry	0	50	100	150	Mean	SEM	*p-value*	Regression
Uric Acid, mg/dL	6.330±1.0981	5.068±0.5014	5.914±1.0086	5.094±0.5842	5.60	0.4045	0.6528	NS
Total Protein, g/dL	3.352±0.2123	3.173±0.1966	3.159±0.2096	2.888±0.1101	3.14	0.0938	0.3981	NS
Creatinine, mg/dL	0.144± 0.0181	0.130± 0.0145	0.117± 0.0156	0.065±0.0794*	0.114	0.0956	0.0078	0.0014⁎⁎
Globulin, g/dL	1.393±0.3851	1.643±0.1478	1.424±0.3117	1.535±0.1690	1.50	0.1268	0.9115	NS
Albumin, g/dL	1.959±0.2393	1.530±0.1508	1.735±0.2367	1.353±0.1631	1.64	0.1061	0.2113	NS
GGT, U/L	24.21± 3.94	27.44± 2.04	23.21± 6.22	21.22± 6.88	24.02	2.4137	0.8564	NS
ALP, U/L	2544±254	2747±342	3612±863	2267±164	2792.36	251.9069	0.2748	NS
ALT, U/L	7.460±3.1692	6.848±0.6975	5.431±1.0723	4.772±0.4410	6.13	0.8281	0.6755	NS
AST, U/L	296.6 ± 44.4	433.6 ± 117.1	365.3 ± 34.2	405.0 ± 20.6	375.13	32.3834	0.5033	NS
Glucose, mg/dL	200.0 ± 29.1	193.8 ± 35.5	198.5 ± 8.0	152.9 ± 9.5	186.29	11.7791	0.4671	NS
Cholesterol, mg/dL	106.4 ± 3.8	89.91± 5.56	96.74± 4.06	92.76±10.21	96.44	3.2808	0.3268	NS
HDL, mg/dL	127.2 ± 7.6	81.55±11.65*	81.20± 6.09*	77.84± 4.02*	91.95	5.9068	0.0010	0.0006*
LDL, mg/dL	55.68± 7.83	60.32± 9.82	40.82± 5.64	38.19± 5.18	48.75	4.0106	0.1284	NS
Triglycerides, mg/dL	270.1 ± 10.1	250.2 ± 27.2	281.8 ± 13.5	239.4 ± 20.7	260.39	9.5257	0.4150	NS
VLDL, mg/dL	54.02± 2.01	50.04± 5.43	56.36± 2.69	47.88± 4.15	52.08	1.9051	0.4150	NS

⁎ Statistical difference (Dunnett test).

⁎⁎ Linear effect. ***Quadratic effect. Linear Effect: (Creatinine: *y*= −0.5008x + 151.36, R2= 0.8777); (HDL: *y*= −0.2969x + 114.21, R2= 0.6615). ALT: Alanine Aminotransferase; AST: Aspartate Aminotransferase; ALP: Alkaline Phosphatase; GGT: Gamma Glutamyl Transferase; HDL: High Density Lipoprotein; LDL: Low Density Lipoprotein; VLDL: Very Low Density Lipoprotein. SEM: Standard Error of the Mean. NS: Not significant. g: Gram; mg: Milligram; dL: Deciliter; U: Unit; L: Liter.

### *Carcass and organ yield*

**Fig. 12:**
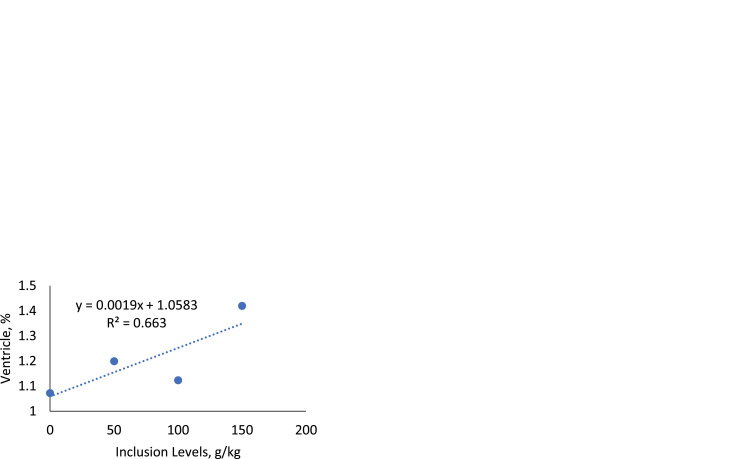
Linear effect of the ventricle of birds fed with SC.

There was an effect of the inclusion (*P* = 0.0077) of SC in the diets on the relative weight of the ventricle (Table 11, Fig. 12) showing an increasing linear effect (*P* = 0.0040). As for the other organ variables (Table 11), and for carcass yield (Table 12), there was no significant effect for the variables analyzed.

**Table 11:** Average values of relative weight of organs and intestine length of broiler chickens fed increasing levels of sesame cake.

	Inclusion Levels (g/kg)							
Organs (%)	0	50	100	150	Mean	SEM	*p-value*	Regression
Spleen	0.101 ± 0.0189	0.090 ± 0.0099	0.088 ± 0.0166	0.083 ± 0.0085	0.0906	0.0067	0.8297	NS
Bursa	0.118 ± 0.0160	0.161 ± 0.0261	0.127 ± 0.0131	0.106 ± 0.0073	0.1281	0.0091	0.1663	NS
Intestine, length (cm)	172.2 ± 5.4	186.0 ± 6.5	184.8 ± 4.6	173.2 ± 12.1	179.05	3.8289	0.4578	NS
Heart	0.387 ± 0.0256	0.492 ± 0.0398	0.422 ± 0.0402	0.439 ± 0.0665	0.4354	0.0226	0.4538	NS
Liver	1.427 ± 0.1027	1.489 ± 0.0817	1.562 ± 0.0915	1.415 ± 0.0846	1.4734	0.0436	0.6529	NS
Total fat	0.850 ± 0.1510	0.806 ± 0.0966	0.655 ± 0.0719	0.760 ± 0.1069	0.7679	0.0533	0.6378	NS
Ventricle	1.072±0.042	1.199±0.088	1.123±0.059	1.420±0.061*	1.2031	0.0425	0.0077	0.0040⁎⁎
Pancreas	0.153 ± 0.0282	0.167 ± 0.0104	0.172 ± 0.0111	0.183 ± 0.0114	0.1688	0.0082	0.6691	NS
Proventricle	0.221 ± 0.0556	0.294 ± 0.0155	0.346 ± 0.0548	0.306 ± 0.0286	0.2921	0.0220	0.2471	NS
Thymus	0.165 ± 0.0129	0.277 ± 0.0743	0.188 ± 0.0287	0.247 ± 0.0409	0.2193	0.0232	0.3081	NS

⁎ Statistical difference (Dunnett test).

⁎⁎ Linear effect. ***Quadratic effect. Linear Effect (*y* = 0.0019x + 1.0583, R² = 0.663). SEM: Standard Error of the Mean. NS: Not Significant.

**Table 12:** Average carcass yield values of broiler chickens fed with the inclusion of increasing levels of sesame cake.

	Inclusion Levels (g/kg)							
Yield	0	50	100	150	Mean	SEM	*p-value*	Regression
Carcass	78.59 ± 1.02	78.27 ± 1.11	79.12 ± 1.21	77.47 ± 0.72	78.36	0.2517	0.2694	NS
Wing	8.88 ± 0.37	9.12 ± 0.20	8.96 ± 0.22	8.74 ± 0.12	8.93	0.1160	0.7420	NS
Thigh + drumstick	27.26 ± 0.45	27.28 ± 0.85	27.79 ± 0.47	28.07 ± 0.10	27.60	0.1507	0.6551	NS
Back	22.32 ± 0.78	23.73 ± 0.60	23.89 ± 1.13	23.92 ± 0.78	23.46	0.4177	0.4997	NS
Breast	41.24 ± 0.95	39.71 ± 0.91	39.48 ± 1.16	39.05 ± 0.87	39.87	0.4868	0.4388	NS

*Statistical difference (Dunnett test).

^⁎⁎^Linear effect.

^⁎⁎⁎^Quadratic effect.

SEM: Standard Error of the Mean. NS: Not Significant.

### Densitometry, Seedor index, and mineral composition of the tibia

The inclusion of sesame cake did not influence the relative weight of the chickens' tibia. The Seedor index did not show a significant difference between the groups (*P* = 0.0836) and for the bone densitometry values (*P* > 0.05) (Table 13). The results showed that the inclusion of sesame cake had no effect on the bone mineralization of the birds.

**Table 13:** Average values of calcium and phosphorus, relative weight, Seedor index, and bone densitometry in different regions of the tibia of broilers fed increasing levels of sesame cake.

Tibia	Inclusion Levels (g/kg)							
	0	50	100	150	Mean	SEM	*p-value*	Regression
Relative weight of the tibia	0.731 ± 0.024	0.775 ± 0.014	0.697 ± 0.068	0.730 ± 0.027	0.733	0.0191	0.5857	NS
Seedor index (mg/mm)	37.15 ± 0.96	36.40 ± 1.11	38.04 ± 1.26	33.69 ± 1.25	36.32	0.6469	0.0836	NS
Calcium (g/100 g)	34.22 ± 1.36	26.82 ± 1.28*	27.25 ± 1.55*	21.63 ± 0.89*	27.48	1.1857	<0.0001	<0.0001⁎⁎
Phosphorus (g/100 g)	11.67 ± 0.55	11.56 ± 0.58	13.08 ± 0.73	10.32 ± 0.42	11.657	0.3482	0.0320	0.0369⁎⁎⁎
Ca:P ratio	2.567 ± 0.117	2.187± 0.085*	2.084±0.015*	2.097±0.011*	2.234	0.0562	0.0007	0.0003⁎⁎
Bone densitometry								
Distal	732.7 ± 39.8	834.6 ± 28.9	882.8 ± 58.0	767.8 ± 54.1	804.47	25.2211	0.1440	NS
Medial	1180 ± 21	1201 ± 35	1170 ± 44	1129 ± 44	1170.03	18.0388	0.5810	NS
Proximal	814.5 ± 30.3	842.2 ± 25.0	797.9 ± 27.5	858.0 ± 11.0	828.15	12.4975	0.3393	NS

⁎ Statistical difference (Dunnett test).

⁎⁎ Linear effect.

⁎⁎⁎ Quadratic effect. Linear Effect: (Calcium: *y* = −0.0747x + 33.081; R² = 0.8699); (Ca:P Ratio: *y* = −0.003x + 2.4607; R² = 0.7415). Quadratic Effect (Phosphorus: *y* = −0.0003×2 + 0.0347x + 11.375; R² = 0.5431; Maximum Point: 57.83 g/kg). SEM: Standard Error of the Mean. NS: Not Significant.

Despite this, calcium concentration was significantly lower in the groups with inclusion of the sesame cake (*P* < 0.0001) (Table 13, Fig. 9). At levels of 50, 100, and 150 g/kg, sesame cake differed significantly from the control diet, with values decreasing as inclusion increased. Regression analysis indicated a decreasing linear effect (*P* < 0.0001) on calcium concentration with the increasing of the sesame cake.

**Fig. 13:**
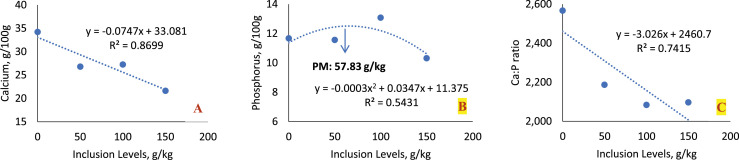
Linear effect of calcium (A) and quadratic effect of phosphorus (B), and calcium ratio (C) in the tibia of birds fed with SC.

A significant difference was observed in phosphorus concentration (*P* = 0.0320) (Table 13, Fig. 13). Regression analysis indicated a quadratic effect (*P* = 0.0369) with a maximum point at the level of 57.83 g/kg, with intermediate levels of sesame cake presenting a higher phosphorus concentration in the tibia, while the lowest and highest levels had a significant reduction.

The calcium:phosphorus ratio (Ca:P) was significantly affected by the inclusion of sesame cake (*P* = 0.0007) (Table 13, Fig. 13). The Ca:P ratio was significantly lower in the groups with the inclusion of sesame cake, especially at the levels of 50, 100, and 150 g/kg, when compared to the control diet. Regression analysis showed a decreasing linear effect (*P* = 0.0003), indicating that increasing sesame cake levels reduced the ratio between these minerals.

### Return on investment (ROI)

**Fig. 14:**
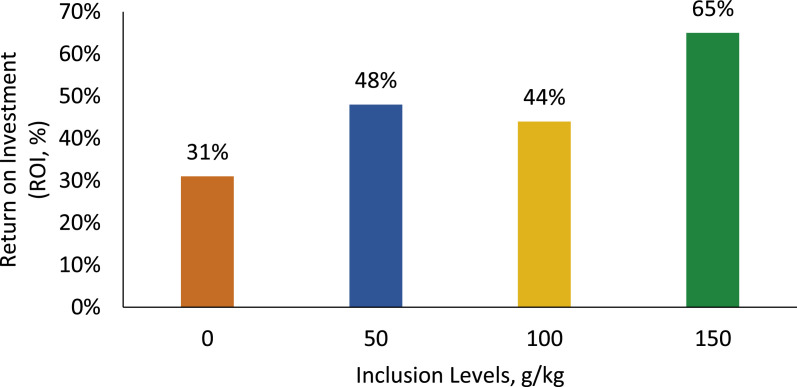
Return on investment comparing diets with and without the addition of SC.

According to the ROI, the percentage of return on investment for birds fed the reference diet was 31 %. When adding sesame cake, the return increased gradually: 48 %, 44 %, and 65 % for inclusions of 50, 100, and 150 g/kg, respectively (Table 14, Fig. 14).

**Table 14:** Phase, total, and per bird feed values. Return on investment (ROI), economic viability.

	Inclusion levels (g/kg)			
Age/Stage	0	50	100	150
	Values by Phase (R$)			
1-7 Days	26.02	24.84	22.09	21.00
8-21 Days	216.29	197.98	194.28	164.64
22-35 Days	423.31	376.70	378.47	329.90
35-42 Days	247.76	219.48	220.11	196.26
Values				
Total feed value	913.38	819.00	815.76	711.80
Sales value of birds (kg)	5.5	5.5	5.5	5.5
Average weight of birds (kg)	3.639	3.663	3.570	3.564
Total sales value of birds (kg)	1,200.87	1,208.79	1,178.10	1,176.12
Investment (feed)	913.38	819.00	815.76	711.80
ROI (%)	31 %	48 %	44 %	65 %

ROI (Return on Investment) = (revenue – cost / investment).

## Discussion

Sesame cake (SC), known for its nutritional richness, had an impact on the zootechnical performance, nutrient metabolizability, bone mineral deposition, blood variables, plantar pad injury score, and ventricle weight of the chickens. It was noted that the inclusion of 100 g/kg of SC resulted in a lower performance in the 1-7-day phase, which may be associated with the lower bioavailability of nutrients, especially calcium.

Calcium oxalate, an antinutritional and poorly absorbed compound that is present in SC in high concentration (1.04 mg/100 g of SC) (Fadimatou et al., 2024), can interact with dietary components and form insoluble complexes in the gastrointestinal tract, reducing the absorption of this essential mineral (Torcello-Gómez et al., 2018). This interaction can affect lipid digestion by preventing efficient lipid emulsification (Mulet-Cabero and Wilde, 2021), reducing the availability of energy and nutrients.

The formation of calcium soaps in the intestine is a factor that may have influenced lipid digestibility, and consequently, energy metabolizability. Torcello-Gómez et al. (2018) report that free fatty acids are more likely to interact with calcium, forming insoluble complexes that are not absorbed. The lipid composition of SC, rich in free fatty acids (Arab et al., 2022), may have contributed to this phenomenon, reducing the efficiency of energy absorption. Furthermore, the late maturation of the digestive tract of birds in the neonatal phase may have increased this effect (Rodriguez-Sanchez et al., 2019).

Despite the effects on nutrient metabolizability, the growth and finishing phases did not have a significant impact on zootechnical performance, although there was a reduction in nutrient metabolizability. This may be related to the greater adaptability of the birds' metabolism in more advanced stages of development, in addition to the lower relative requirement for calcium in these phases. Furthermore, the greater inclusion of SC resulted in lower density diets, making the diets more voluminous, which may have influenced the reduction in effective nutrient intake.

Greater inclusion of SC increased ventricle weight, possibly due to the greater volume of the diet, stimulating the mechanical function of this organ (Kiarie e Mills, 2019). Studies indicate that higher volume diets promote greater retention of digesta in the gizzard, improving the grinding and digestibility of food (Zaefarian et al., 2016; Abd El-Wahab et al., 2020).

Calcium and phosphorus play an essential role in energy metabolism (Voorrsluijs et al., 2024) and in the physiological homeostasis of birds (Ribeiro et al., 2024). Calcium is crucial for muscle contraction, neuronal transmission, and enzyme activation, in addition to being directly involved in glycolysis and oxidative phosphorylation, impacting ATP synthesis (Voorrsluijs et al., 2024). The reduction in calcium bioavailability due to the presence of oxalates can affect these processes, reducing the efficiency of energy production and compromising the initial growth rate. Phosphorus, in turn, is essential in the formation of membrane phospholipids, participates in nucleotide synthesis, is an essential component of ATP, and is crucial for the regulation of acid-base homeostasis and bone metabolism (Nicholls et al., 2023). The quadratic effect observed in phosphorus deposition may be related to compensatory mechanisms of absorption and excretion of this mineral.

Bone mineralization analysis revealed that the inclusion of SC reduced calcium levels in the tibia, compared to the control group. Phosphorus, in turn, showed a quadratic effect, with a peak deposition in the diet containing 100 g/kg of SC. The calcium/phosphorus ratio decreased linearly with the increase in SC inclusion, reflecting a possible imbalance in mineral homeostasis, although the 2:1 ratio was maintained. However, bone densitometry indicated that there was no significant difference in bone mineral density, suggesting that the physiological mechanisms of the birds compensated for the lower bioavailability of calcium through regulatory mechanisms, such as greater intestinal absorption.

The calcium content found in the tibia of birds fed the control diet was similar to that reported by Imari et al. (2020), who observed values ranging from 30.79 to 35.73 g/100 g when evaluating diets with different levels of calcium and phosphorus for broilers. The calcium content in the tibia of birds fed the diet containing SC showed values close to those described by Díaz-Alonso et al. (2019), who recorded variations between 26.86 and 30.59 g/100 g when testing diets with different levels and ratios of calcium and available phosphorus. The result observed in this study for the calcium content in the tibias of chickens fed SC is within expectations, considering the presence of calcium oxalate, which reduces the bioavailability of this mineral. Even so, the values obtained are compatible with those found in literature.

Regarding phosphorus, Díaz-Alonso et al. (2019) reported values between 16.93 and 17.13 g/100 g, while Imari et al. (2020) observed a greater range, with values from 14.64 to 17.86 g/100 g. In the present study, the phosphorus content in the tibias of chickens fed with SC was considerably lower than the values described in literature. However, the calcium:phosphorus ratio (Ca:P) remained within the recommended range (2:1), which contributed to the fact that the bone densitometry of birds fed SC did not show reduced values.

Sesame seeds have a high concentration of bioactive compounds with anti-inflammatory and antioxidant properties (Sharma et al., 2021). These compounds are mostly present in the oil fraction of the seed (Bopitiya and Madhujith, 2015), which suggests that SC, because it contains a significant amount of ether extract, also presents high concentrations of these substances.

In this context, the anti-inflammatory compounds present in SC may have contributed to the improvement of the pododermatitis score in broilers, reducing the size of the lesions. In an *in vitro* study, Deme et al. (2018) demonstrated that methoxyphenol derivatives present in sesame oil exhibit potent anti-inflammatory properties, as do lignans, tocopherols, and phytosterols, which interact synergistically to combat inflammation and promote a more efficient anti-inflammatory response.

In addition to the effects on bird health, SC influenced serum creatinine levels, promoting a significant reduction in this biomarker. The values obtained in our research are in agreement with those presented by Kim et al. (2015), who reported variations between 0.09 and 0.25 mg/dL when working with broilers. Creatinine, a metabolite of creatine present in the blood, is filtered by the kidneys and excreted in the urine. Its serum levels are indicators of renal function, and high concentrations may suggest muscle dysfunction or injury, especially when associated with variations in ALT and AST levels (Delanaye et al., 2017). Thus, the reduction in serum creatinine levels with the use of SC suggests a protective effect on the kidneys of broilers.

Sesame byproducts tend to be rich in antioxidants, which contribute to the reduction of serum creatinine levels by increasing the activity of serum antioxidants such as superoxide dismutase, glutathione peroxidase, and catalase, which fight free radicals and reduce the level of malondialdehyde (El-Tahan et al., 2019; Ye et al., 2020). These benefits are mainly attributed to sesamin, a lignan predominant in the oily fraction of sesame, known for its richness in gamma-tocopherol and potent antioxidant action (Gharby et al., 2017).

Regarding lipid metabolism, although SC is rich in unsaturated fatty acids, a reduction in serum HDL levels was observed. This can be explained by the increased concentration of circulating free fatty acids. In the initial phase (1-21 days), lower inclusions of SC favored bird performance, possibly due to a lower concentration of circulating free fatty acids, ensuring an adequate energy supply. However, in the following phases (1-35 and 1-42 days), the inclusion of SC did not compromise performance, possibly due to the presence of soybean oil in the diets, which improves digestive transit time and nutrient absorption.

Another relevant factor is the role of sterols and sesamin in intestinal cholesterol absorption. These compounds can reduce cholesterol absorption by inhibiting the Niemann-Pick C1-Like 1 (NPC1L1) transporter and decreasing cholesterol conversion in intestinal cells (Liang et al., 2015; Wang et al., 2023). However, in our research, no changes were observed in the levels of triglycerides, total cholesterol, and LDL, a result consistent with the findings of Salavati et al. (2021), who did not detect changes in these parameters in broilers fed bioactive peptides from sesame flour. Furthermore, the serum HDL values found are similar to those reported by Carvalho et al. (2020), which ranged from 89.22 to 113.95 mg/dL for broilers.

Regarding the immune response, the progressive inclusion of SC in the diet resulted in a reduction in white blood cell counts, although these values remained within the normal range. This reduction is interpreted as indicative of a lower immune challenge in the birds of the present study, potentially reflecting an improvement in their immune response. Khorrami et al. (2018) demonstrated that sesame essential oil and its phenolic compounds, such as sesamol, have immunomodulatory properties, suppressing the cellular immune response through the regulation of Th2 responses and the modulation of the pro-inflammatory functions of macrophages and dendritic cells. These effects are directly related to the antioxidant and anti-inflammatory properties of the phenolic compounds present in the sesame oil fraction.

In comparison with the data from Carvalho et al. (2020), the values of leukocytes, heterophils, and lymphocytes reported by these authors for broilers (23.00 × 10³/mm³; 10,418 U; 9,188 U, respectively) were higher than those obtained in the present study for heterophils and lymphocytes, but similar in relation to total leukocytes. When evaluating hematological parameters in broilers, Soares et al. (2020), reported values of leukocytes, heterophils, lymphocytes, and monocytes of 15.25 × 10³/mm³; 3,310 U; 9,340 U, and 1,610 U, respectively, demonstrating similarity only with the values of leukocytes and heterophils observed in this study. Variations between results can be attributed to environmental, dietary, and hormonal factors, which influence the immune response of birds (Soares et al., 2020). Despite these differences, the observed values remain within the physiological parameters considered normal, with no evidence of compromising the health of the birds.

Finally, the inclusion of SC in the diet showed a positive financial return, especially with the inclusion of 150 g/kg, the level at which the best return on investment (ROI) was observed, compared to other treatments and the control group. This result reinforces the economic viability of SC, particularly for small and medium-sized rural producers, when compared to soybean meal. At the time of the study, the cost of SC was R$ 1.80/kg, while soybean meal cost R$ 3.86/kg, making SC an economically advantageous alternative.

The obtained results demonstrate the potential of SC as a nutritional and functional ingredient in broiler feed. Despite the limitations in its inclusion in the initial phases, due to the presence of antinutritional factors and energy availability, its use showed benefits in both the final cumulative performance and the health of the birds. In addition, the nutraceutical effect of SC was evident, promoting improvements in the immune system and reducing the incidence of pododermatitis. Thus, SC is a promising alternative for poultry farming, favoring both productivity and animal welfare.

## Conclusion

The inclusion of sesame cake in the diet of broilers demonstrated economic and zootechnical viability, providing a greater financial return without compromising the final performance of the birds. Although levels above 56.85 g/kg reduced performance in the initial phases, the inclusion of up to 150 g/kg did not negatively affect performance in the total period of 1 to 42 days, nor did it negatively affect bone densitometry parameters, as well as providing a lower incidence of pododermatitis, which is an indicative of improvement in the immune response. Therefore, SC can be used up to 150 g/kg for broiler chickens. As SC is an alternative feed, it should be further studied in order to better understand its nutraceutical benefits.

## CRediT authorship contribution statement

**Dayane Albuquerque da Silva:** Writing – review & editing, Writing – original draft, Project administration, Methodology, Investigation, Formal analysis, Data curation, Conceptualization. **Maria do Carmo Mohaupt Marques Ludke:** Writing – review & editing, Writing – original draft, Visualization, Validation, Supervision, Resources, Project administration, Methodology, Investigation, Funding acquisition, Formal analysis, Data curation, Conceptualization. **Jorge Vitor Ludke:** Writing – original draft, Software, Methodology, Data curation, Conceptualization. **Lilian Francisco Arantes de Souza:** Formal analysis, Data curation, Conceptualization. **Mércia Rodrigues Barros:** Writing – original draft, Software, Data curation, Conceptualization. **Ana Carolina Ferreira dos Santos:** Formal analysis, Data curation, Conceptualization. **Fabiano Séllos Costa:** Formal analysis, Data curation, Conceptualization. **Sérgio Peres:** Resources, Methodology, Formal analysis, Conceptualization. **Camila Guedes Valadares:** Investigation, Formal analysis, Data curation, Conceptualization. **Arlei Coldebella:** Software, Conceptualization. **Esterfani Pereira da Silva:** Formal analysis, Data curation, Conceptualization. **Lucas Rannier Ribeiro Antonino Carvalho:** Writing – original draft, Data curation, Conceptualization. **Apolônio Gomes Ribeiro:** Formal analysis, Data curation, Conceptualization.

## Disclosures

The authors declare that they have no other conflicts of interest. This work was supported by Karolinska Institutet through an institutional publication agreement. The funding covered the open access publication fees.
